# Compulsory Psychiatric Treatment at Home in the Netherlands: A Case Report

**DOI:** 10.1155/crps/4088726

**Published:** 2026-02-23

**Authors:** D. A. de Waardt, G. A. M. Widdershoven, M. H. C. M. Laan, R. Favie, C. L. Mulder

**Affiliations:** ^1^ Department of Psychiatry, ETZ Hospital (Elisabeth-TweeSteden Ziekenhuis), Tilburg, Netherlands; ^2^ Department of Psychiatry, Epidemiological and Social Psychiatric Research Institute (ESPRi), Erasmus University Medical Center, Rotterdam, Netherlands, erasmusmc.nl; ^3^ Department of Ethics, Law and Humanities, Amsterdam University Medical Centers (location VUmc), Amsterdam, Netherlands, uva.nl; ^4^ GGNet, Warnsveld, Netherlands; ^5^ Breburg Psychiatric Institute, Tilburg, Netherlands; ^6^ Ministry of Defense Healthcare Organisation, Den Bosch, Netherlands; ^7^ Parnassia Psychiatric Institute, Rotterdam, Netherlands

## Abstract

An important aim of the new mental health act implemented in the Netherlands in 2020 was to shift the focus from hospitalization to treatment. The act provides an option for patients’ compulsory treatment at home (CTH). Unlike compulsory community treatment (CCT), which allows mental health workers to hospitalize patients involuntarily if they do not comply with treatment. CTH is not provided elsewhere in the world. This case report concerns a patient who, to avoid hospitalization, received CTH in the form of compulsory medication and was able to recover at home. Retrospectively, she and the mental health team both positively evaluated this use of CTH. The parties involved all concluded that CTH restricted the patient’s autonomy less than hospitalization, it did not influence the therapeutic relationship, and the CTH could be delivered in a way that it did not compromise participants’ safety. This case report shows that, in some situations, CTH can avoid hospitalization. Care should nonetheless be taken to assess its appropriateness, to discuss any preconditions, and to evaluate the use of compulsory treatment in dialog with the patient and significant others.

## 1. Introduction

The Compulsory Mental Healthcare Act was implemented in the Netherlands in 2020, replacing the former act from 1994, the Special Admissions to Psychiatric Hospitals Act (BOPZ). The implementation of this new act marked a shift in the approach to compulsory mental healthcare in the Netherlands. The focus shifted from hospital admission to the provision of treatment, regardless of the setting in which the treatment occurs. The Compulsory Mental Healthcare Act includes provisions for compulsory community treatment (CCT) and compulsory treatment in the patient’s home.

Before the introduction of the new Compulsory Mental Healthcare Act, CCT was already an option under the former legislation. However, if patients resisted treatment, involuntary treatment could only be provided in a hospital setting. The new mental healthcare act introduced the option of compulsory treatment at home (CTH), broadening the scope of treatment at home to avoid hospitalization. Working on the basis of a court order for CTH, mental healthcare professionals are permitted to use specified forms of compulsion in the patient’s home. For example, they may compel a patient to take their medication or search their home for drugs. If a patient resists taking medication, the law also allows for the use of physical restraint in the patient’s home. By introducing the possibility of CTH, the Dutch government intended to enable healthcare workers to treat more patients at home and thus fewer patients in hospital. To the best of our knowledge, CTH has not been implemented elsewhere in the world.

The Compulsory Mental Healthcare Act provides for two types of orders under which CTH can be used. The first concerns acute situations in which a patient is at serious risk of harm or disadvantage. Following an assessment by an independent psychiatrist, the mayor of the patient’s municipality may issue an order lasting for a maximum of 3 days. After these 3 days, if necessary, a judge can then extend this period by 3 weeks.

The second type of order concerns situations in which disadvantage is imminent but less acute. In such cases, a judge bases their ruling on a care plan, ideally drawn up by the patient, their significant others, and the treating psychiatrist, and supported by an additional report by a second, independent psychiatrist. This care plan allows patients to state their preferences with regard to compulsory care. This second type of order applies to periods of up to 6 months [1]. CTH can be used under both types of order, the difference being that the second type of order has a longer duration and can be used in less acute situations.

The act also describes 11 different forms of compulsory care that can be used under these two types of orders (Table 1). Per order, the mayor or judge decides which forms of compulsory care will be included and can thus be used if necessary. These forms of compulsion are also described in the care plan. The psychiatrist in charge of treating the patient then decides which forms will be used in situations that require the terms of the order to be implemented. Various forms of compulsory care can be used simultaneously, such as compulsory medication and admission to hospital. It is also possible for patients to draw up a “zelfbindingsverklaring,” an advance directive, which is a legal document that states what types of compulsory treatment a patient would prefer.

**Table 1:** The 11 forms of involuntary treatment specified in the Dutch Compulsory Mental Healthcare Act.

a. Administering fluids, nutrition, and medication. Performing medical checks or other medical actions or interventions to treat the mental health disorder or any physical disorder caused by it
b. Limiting the patient’s freedom of movement
c. Confining the patient
d. Monitoring the patient
e. Examining the patient’s clothes or performing a physical examination
f. Searching the patient’s home, or the place where they are staying, for dangerous items and substances that could influence their behavior
g. Checking for the presence of substances that influence behavior
h. Restricting the patient’s freedom or ability to arrange their own life
i. Limiting the right to visitors
j. Admitting a patient to the hospital
k. Taking away a patient’s freedom by bringing them to a place that is fit for temporary stay

Internationally, the use of compulsory treatment in a patient’s home is a controversial and much‐debated subject [2]. Before CTH was implemented in the Netherlands, stakeholders were concerned about the ethical and practical challenges inherent in such treatment [3].

CTH may be less restrictive than involuntary hospitalization and can be seen as an alternative that interferes less with human rights and dignity than compulsory admission to a psychiatric facility. However, CTH can also involve a significant restriction of freedom and may undermine the therapeutic relationship. It effectively extends the use of coercion into the community, potentially making it more challenging to protect the rights of patients in nonhospital settings.

Before the Compulsory Mental Healthcare Act came into force, however, the Dutch psychiatric association issued a guideline advising against the use of physical restraint in a patient’s home (Dutch Psychiatric [4]). This recommendation was based on safety considerations. In recent years, however, a gradual shift has been observed, with mental healthcare professionals becoming more open to the use of physical restraint in the patient’s home [5].

This case report aims to illustrate the use of CTH in practice and to reflect on the ethical and practical challenges referred to in the literature.

### 1.1. Setting

The patient described had been receiving compulsory care from an outpatient team, an Assertive Community Treatment (ACT) team that provides the outreach‐based mental healthcare for people living in the community. The team consisted of psychiatric nurses, a psychologist, a social worker, a doctor in training to become a psychiatrist, and a psychiatrist. The patient’s psychiatrist supplied the information in this case report, drawing on her own experience with the patient, the team evaluation, and relevant information from the patient’s file.

### 1.2. Ethics

After reviewing the study proposal, Medical Ethics Review Board Brabant, the committee that assesses ethical aspects of scientific research, concluded that the study did not entail clinical research with human subjects as defined in the Medical Research Involving Human Subjects Act, and thus that formal approval was not necessary. However, participation was restricted solely to patients who were deemed competent to give their approval. The patient gave informed consent to use an anonymized description of her situation for this case report.

## 2. Case

The case involved a 62‐year‐old woman who had been diagnosed with a schizoaffective disorder 8 years earlier. At the time of the study, she had been receiving care from her current mental healthcare institution for 7 years. During this period, she had been hospitalized involuntarily twice (7 and 4 years previously); on both occasions, she had recovered soon after starting antipsychotic medication. After the first hospitalization, she established contact with her current ACT team.

At the time of the study, she was living in a house with three other patients. In this house, they each have their own bedroom but share other facilities. Healthcare staff is present in the daytime and can be reached by telephone at night. Our patient was afraid of mental healthcare, the government, and her housemates and frequently showed signs of being paranoid. However, when she was doing well, she did not avoid her ACT team. Her autonomy and independence were very important to her, and she wished to live on her own, initially with support from her mental healthcare workers. In the long run, she hoped to live independently. She had no social support beyond mental healthcare workers and the staff in her supported accommodation. She had no contact with friends or members of her family. She corresponded with two friends she had never met in person.

In the past, she had gotten into trouble due to her illness and had become homeless. She is still convinced that the loss of her house was unlawful and was the fault of drug‐abusing neighbors. This experience still frightens her and remains a topic of conversation. When she lost her house, she was also hospitalized involuntarily. This stay in the hospital was adverse for her; she felt that her freedom and autonomy were taken away from her. Nevertheless, during her hospital stay, she was cooperative, and she later reported that she was treated well.

In 2024, after reducing her medication and following the rejection of her request to move to accommodation offering more independence, she again developed psychotic symptoms. She became paranoid towards her housemates, care staff, and mental healthcare workers. She did not trust their intentions and refused treatment. She became hostile and verbally aggressive towards the support staff in her supported accommodation, as well as towards her neighbors and mental healthcare workers. She avoided contact with the support staff and stayed in her room all day. Her aggressive behavior scared her housemates and prevented the support staff from caring for her as they had. Despite her hostility, the support staff and mental healthcare team—who were aware of her rapid improvement during previous hospitalizations and her continued anger about these hospitalizations—considered it preferable to try to avoid another hospital admission by providing care at home.

At that point, although the patient was unable to make choices about her treatment plan, she did say that she did not want to be hospitalized. The mental healthcare workers therefore decided to apply for an emergency order for involuntary medication‐based treatment at home. As she was being aggressive towards those in her supported accommodation and towards strangers outside her house, her treatment team deemed the situation to be acute and applied for the mayor to issue an emergency order. Accordingly, the mayor approved the start of CTH.

After the order had been granted, it was explained to her that she would receive intramuscular medication at home, and medication at home was started. Treatment involved the administration of intramuscular slow‐release zuclopenthixol, as she refused oral administration and had responded well to this type of medication before. Two members of the support staff and two members of the mental healthcare team (a nurse and the psychiatrist who had applied for the CTH order) were present the first time she received this treatment. The patient was willing to lie on her bed and make herself comfortable before the nurse administered the medication. While the psychiatrist held her hand to comfort her, she accepted the nurse’s intervention. However, this was because she knew she had to undergo the treatment since she had a court order, not because she willingly accepted the use of medication.

To ensure the safety of all people involved and to prevent the patient from running away or from reacting in an unforeseen way, the mental healthcare team had also asked the police to be present. To respect the patient’s privacy, the police officers remained in the hallway, and it did not prove necessary to call them into the bedroom. If the patient had resisted, the officers could have come in to restrain her to facilitate the treatment at home, or they could have assisted in the transfer to the hospital if that had been deemed necessary.

The compulsory administration of intramuscular medication at home ended after the second home visit, on which medication was administered, when the patient declared her willingness to take oral medication. Since then, despite the mental healthcare team’s application for a long‐term court order, compulsory treatment has not been necessary. The patient recovered from her psychosis at home, and her situation stabilized. The mental healthcare team visited her frequently—twice a week at first, but by the time of writing, this had been reduced to one visit every 4–6 weeks. The mental healthcare team also remained available to help the support staff at the supported accommodation.

According to standard procedure, and in addition to evaluating the patient’s response after administering medication, the mental healthcare team also assessed the compulsory treatment intervention as a whole. The patient was present at the final evaluation of her CTH. She expressed satisfaction with the way events had unfolded and was glad that she had not had to leave her living environment. “I think hospitalization is a very drastic measure,” she said. “There are other solutions, such as an injection at home. Hospitalization does me more harm than good.” When she was asked about the presence of the police, she said she could not recall it.

Looking back, the mental healthcare workers said that it had been a logical decision to administer the treatment at home rather than in a hospital. Having carried out CTH before with other patients in less acute situations, they already had relevant experience. Knowing that the patient was still angry about her previous involuntary hospitalizations, they were glad that they did not have to jeopardize the fragile therapeutic relationship by repeating the experience. Similarly, the support staff from the supported accommodation were pleased that they had been able to continue care in a more complex situation. In a separate evaluation, the police officers involved said that they saw their presence in such situations as part of their duty and knew that such assistance could be asked of them.

## 3. Discussion

As CTH is a new option in Dutch mental healthcare and has not been applied internationally, little has been published regarding its practical application. Our case is unique in that it describes both the process and the experiences of stakeholders in a specific instance in which CTH was used.

Before CTH was implemented in the Netherlands, stakeholders raised various concerns, some of which were of an ethical nature, especially regarding the infringement of patients’ autonomy [3]. This concern has also been raised when introducing CCT in, for example, the United Kingdom [2]. However, after the introduction of CTH, focus groups revealed that patients, significant others, and mental healthcare workers expressed more positive views than they had beforehand, suggesting, for example, that CTH could give a patient more autonomy than hospitalization [6].

The compulsory care provided in the case described—intramuscular medication at home in an acute situation—was intended to avoid hospitalization and to restrict the patient’s autonomy as little as possible. Hospitalization was successfully avoided, and the patient later evaluated the intervention positively. Support staff and healthcare professionals were also positive about the extent to which the CTH preserved the patient’s autonomy.

A remaining concern is the potential effect of compulsory care at home on the therapeutic relationship. A survey investigating mental health workers’ expectations of CCT and CHT found that mental healthcare workers feared that CTH might damage the therapeutic relationship [3]. However, this concern was not reported in a subsequent interview study with patients who had received CTH [7]. Similarly, the mental healthcare workers in the present case did not experience any negative impact on their relationship with the patient. Before administering the medication, they feared the patient might become angry, but during and after the intervention they observed that she did not blame them for using compulsory treatment. Nor did she express anger when the treatment was evaluated some weeks later.

Another concern that has been raised regarding treatment at home is that mental healthcare workers may be less able to monitor a patient’s condition than in a hospital setting. Thus, for example, they would be less likely to detect possible side effects of medication [7]. In the present case, however, this did not appear to be a problem, as the patient lived in a supported independent living accommodation, and the mental healthcare workers were in close contact with the relevant support staff.

A further practical issue raised by stakeholders prior to the introduction of the Compulsory Mental Healthcare Act was whether compulsory care could be provided safely in a home setting [3]. The present case shows that the staff were indeed able to administer compulsory medication in a relatively calm and comfortable manner. The team already had experience using CTH and knew the patient and all the stakeholders well. The patient did not resist the administration of the medication and felt supported by her treatment team. The police were kept at a distance; their presence nonetheless indicated that the healthcare professionals could not entirely exclude the risk of unpredictable behavior on the part of the patient and that, if such behavior were to occur, it would create tension. In a focus‐group study involving stakeholders, most patients and significant others opposed police involvement in compulsory care [6].

Not all of the types of compulsory care that are theoretically permitted under the current mental health act are suitable for use at home. The present case demonstrates that compulsory medication and home visits can indeed be executed effectively. We also believe that physical examinations and blood tests can be performed at home. However, we consider all other forms of compulsory interventions at home to be problematic, as they would excessively restrict the patient’s autonomy and could not be performed safely in a home setting.

Providing compulsory treatment, especially in a patient’s home, is a complex and sensitive matter that should be approached with the utmost care. The Dutch Compulsory Mental Health Act specifies four requirements that must be met when compulsory care is provided: efficiency, subsidiarity, proportionality, and safety. Each time a mental healthcare worker decides to use compulsory care, these requirements should be carefully assessed and documented in the patient’s file.

Coercion in the community differs in several important ways from coercion during hospital admission. One distinction is that coercion in the community may be applied when psychiatric symptoms are less severe. For example, mental healthcare workers might use coercive treatment to maintain safety at home, even though coercion may not have been necessary in a hospital. Another distinction lies in the types of intervention that can be used. In a hospital setting, interventions may include preventing a patient from leaving the ward unsupervised or monitoring them on camera. In a home setting, by contrast, coercion may involve restricting a patient’s freedom to arrange their daily life. Moreover, in the community, patients may be subjected to coercive measures for longer periods, as such measures are often used preventatively, whereas in a hospital they are usually applied during an acute episode [8].

Between voluntary treatment and coercion, there is a spectrum of intermediate arrangements. This spectrum encompasses varying degrees of pressure, beginning with persuasion, progressing through interpersonal leverage and inducements, and ending with threats [9]. When a patient is living at home, mental healthcare workers should be particularly aware of this gray area between voluntary and compulsory care [5].

To date, no studies have established which groups of patients would be the most likely to benefit from CTH. Based on an earlier focus‐group study, we theorized that CTH may be particularly useful for patients who already know which types of intervention are helpful if their mental health deteriorates and who have either a support system or care team to help them at home (de Waardt et al., 2025). The present case supports this view: the patient had a good experience with this medication and was supported both by the treatment team and by professionals in the supported accommodation.

In this case, CTH worked well for the patient. However, it remains unclear which patients are most likely to benefit from CTH. Although CTH can promote autonomy and stimulate patients to continue participating in society, it can also limit their freedom. Each case therefore requires careful assessment to determine whether CTH is an appropriate treatment option. Ideally, this should be discussed with patients in advance so that they can express their preferences regarding compulsory care.

## 4. Conclusion

This case shows that CTH can be a suitable treatment option for patients in certain situations, for example, for those wishing to avoid hospitalization. However, it remains uncertain which patients are most likely to benefit from CTH. In this instance, the treatment team was able to draw on its previous knowledge of how the patient had responded to medication, but the supported independent living accommodation also made it possible to care for her at home. Such factors can be helpful when providing CTH.

We recommend advance discussions with patients and significant others about the possibility of CTH should the patient’s mental health deteriorate. If CTH is later implemented, the experience should be evaluated by all stakeholders. Lessons learned can then be used to refine the treatment plan for future care.

## Funding

No funding was received for this manuscript.

## Consent

The patient gave informed consent to use an anonymized description of her situation for this case report. She signed a written informed consent form.

## Conflicts of Interest

The authors declare no conflicts of interest.

## Data Availability

The data that support the findings of this study are available upon request from the corresponding author. The data are not publicly available due to privacy or ethical restrictions.
